# Surface Detection of THC Attributable to Vaporizer Use in the Indoor Environment

**DOI:** 10.1038/s41598-019-55151-5

**Published:** 2019-12-09

**Authors:** Cristina Sempio, Emily Lindley, Jost Klawitter, Uwe Christians, Russell P. Bowler, John L. Adgate, William Allshouse, Lauren Awdziejczyk, Sarah Fischer, Jacquelyn Bainbridge, Mike Vandyke, Rahwa Netsanet, Tessa Crume, Gregory L. Kinney

**Affiliations:** 1 iC42 Integrated Solutions in Systems Biology for Clinical Research & Development, University of Colorado Anschutz Medical Campus, Aurora, CO USA; 2 Department of Orthopedics, University of Colorado Anschutz Medical Campus, Aurora, CO USA; 3 National Jewish Health and University of Colorado Denver, Denver, CO USA; 4 Department of Environmental and Occupational Health, Colorado School of Public Health, University of Colorado Anschutz Medical Campus, Aurora, CO USA; 5 Skaggs School of Pharmacy and Pharmaceutical Science, University of Colorado Anschutz Medical Campus, Aurora, CO USA; 6 Colorado Department of Public Health and the Environment, University of Colorado Anschutz Medical Campus, Aurora, CO USA; 7 Department of Epidemiology, Colorado School of Public Health, University of Colorado Anschutz Medical Campus, Aurora, CO USA

**Keywords:** Patient education, Epidemiology

## Abstract

The number of cannabis users increased up to 188 million users worldwide in 2017. Smoking and vaping are the most common consumption routes with formation of side-stream smoke/vapor and secondhand exposure to cannabinoids has been described in the literature. External contamination of hair by cannabis smoke has been studied but there are no studies on third-hand cannabis exposure due to deposition of smoke or vapor on surfaces. We tested whether cannabinoids could be detected on surfaces and objects in a room where cannabis is vaporized. Surface samples were collected using isopropanol imbued non-woven wipes from hard surfaces and objects. Each surface was swabbed three times with standardized swabbing protocol including three different patterns. Samples were analyzed using LC-ESI-MS/MS in combination with online extraction. THC was detected on 6 samples out of the 15 collected in the study room at quantifiable levels ranging 348–4882 ng/m^2^. Negative control samples collected from areas outside the study room were all negative. We demonstrated that surfaces exposed to side-stream cannabis vapor are positive for THC at quantifiable levels. This study represents a first step in understanding how side-stream cannabis vapor deposits in the environment and potentially results in a tertiary exposure for users and non-users.

## Introduction

Cannabis is the most commonly used recreational drug worldwide with an estimated 188 million users between the ages of 15–64 in 2017, marking an increase of roughly 16% in the decade ending in 2016^1^. Cannabis is still an illicit drug in most countries; however, several nations around the world and 33 states plus the District of Columbia in the U.S. have passed legislation legalizing or decriminalizing medical cannabis use. Recreational use has also been legalized in Uruguay, Canada and in 10 states and the District of Columbia across the U.S. in recent years^1^.

Delta-9-tetrahydrocannabinol (THC) is the active component responsible for the psychotropic effects in humans. THC is present in the plant material as an acidic precursor (THCA) and is converted into THC via decarboxylation upon heating of the cannabis flower^2,3^. Inhalation from smoking or vaporizing (vaping) is the most common consumption method resulting in side-stream smoke/vapor containing cannabinoids^2,4–6^. Current research suggests that secondhand exposure to side-stream smoke/vapor from cannabis consumption may result in detectable levels of cannabinoids in blood, urine, oral fluid or hair samples of cannabis non-user volunteers^7–14^.

In recent years, there has been increased interest in understanding the effects of passive or indirect exposure to side-stream smoke/vapor components from tobacco and illicit drugs. Established literature on tobacco shows that third-hand exposure can occur via contact with house dust and contaminated surfaces^15–17^. Experimental results demonstrate that surfaces can be contaminated by side-stream smoke from cocaine, methamphetamine and opium^18,19^. No research to date has been published regarding the potential contamination of surfaces from side-stream cannabis smoke or vapor. However, Moosmann *et al*. proved external contamination of hair samples in children living in households where cannabis is consumed and they suggest passive transfer by contaminated hands or surfaces as the primary source of contamination^14,20^. Cannabinoid findings in a child’s hair are of concern in forensic cases (i.e. child-custody) and a better understanding of possible mechanisms of external contamination is highly recommended.

Thus, the goal of this study was to determine whether cannabinoids can be quantitatively detected on room surfaces exposed to side-stream cannabis vapor, making these surfaces possible sources for third-hand exposure.

## Methods

The current study leveraged the protocol of an ongoing IRB approved clinical trial investigating the efficacy of cannabis for chronic and experimental pain alleviation (Colorado Multiple Institutional Review Board [COMIRB] 14–1909). All participants to the clinical trial provided written informed consent and the study was conducted in accordance with Good Clinical Practice guidelines and the Declaration of Helsinki. Environmental sampling was performed in between drug administration visits and no human subjects were involved. The double-blind study protocol exposed participants to vaporized whole plant cannabis (approximately 4.5–5.4% THC and a placebo with 0.002% THC) provided by the National Institute on Drug Abuse (NIDA) Drug Supply Program. The clinical trial was conducted in a single drug administration room (27.26 m^3^) on the University of Colorado Anschutz Medical Campus, Clinical Translation Research Center that was equipped with a high efficiency ventilation system that replaces room air in less than two minutes when activated. Each participant in the clinical trial underwent 3 drug administration visits in the room: one session with active vaporized THC cannabis and 2 with placebo cannabis plant material. The blinded study pharmacist prepared the plant material for vaporization in the secured schedule 1 medication room across the hall from the administration site. The plant material was rehydrated within 12 to 24 hours of administration. The pharmacist delivered the prepared product to the administration room and assembled the Volcano Vaporizer. The clinical trial used a Volcano Vaporizer (Storz-Bickel, Tuttlingen, Germany) in which 400 mg of cannabis or placebo was vaporized at 200 °C into an 8 liter balloon^3^. The filtration fan was turned on prior to inflating the balloon and study personnel left the room. A valve on the balloon allowed the participant to interrupt, at will, the inhalation from the balloon and limited the release of vapor into the indoor environment. The subjects inhaled using the Fulton puff method: 5 seconds inhale, 10 seconds hold, 40 seconds to exhale before their next inhalation. Participants were required to inhale 4 puffs from a balloon and then had the option to inhale up to 4 more puffs from a second balloon. Upon completion of administration the study pharmacist expelled the remaining vapor (if any) towards the ceiling ventilation system and returned the used product to the secured schedule 1 medication room for disposal/storage.

### Surface sampling

The objects in the drug administration room included one fixed and one movable table, a reclining chair, a Grooved Pegboard Task assessment and the vaporizer. The room itself had two sealed windows that extend to the ceiling. During our surface sampling period, study personnel cleaned the study area by wiping only the tables at the beginning of each month with Sani-Cloth germicidal disposable wipes, which were imbued with a mixture of ammonium chlorides derivatives (0.5%), water (45%) and isopropyl alcohol (IPA, 55%) (PDI, Orangeburg, NY, USA). For this reason, the protocol to quantify third-hand exposures was implemented over 2 sampling periods: one at the beginning of the month, after the tables had been cleaned, and one at the end of the month after 6 study visits took place. Since the study staff cleaned only the tables, the other sampled surfaces would represent THC accumulation over time.

Furthermore, due to the double-blind nature of the study, we could not know whether a specific study visit used active or placebo cannabis, so we focused on the possible accumulation of THC on surfaces after repeated administrations of both active and placebo cannabis. However, we were able to estimate the number of active visits that took place in the administration room based on the number of subjects that completed all the three visit of the study. At the time of the first sampling, 44 study visits had occurred in the drug administration room and 12 subjects completed the study allowing for a minimum of 12 active cannabis administrations to a maximum of 18. At the time of the second sampling, 6 additional visits had occurred resulting in 50 total study visits in the room and 13 subjects completed the study allowing for a minimum of 13 active cannabis administration sessions to a maximum of 21.

Due to the lipophilicity of THC and other cannabinoids, samples were collected using non-woven wipes imbued with a mixture of water and isopropyl alcohol (IPA) (30:70, v/v) (Novaplus, Irving, TX, USA). Samples were obtained at the two sampling periods from surfaces that would not be damaged by IPA. In the first sampling period, samples were collected from the tables, the floor, the chair seat, the Grooved Pegboard Task assessment, the door knob, the vaporizer and the window bar. During the second sampling period, samples were collected from the tables, the windowsill and the Grooved Pegboard Task assessment. Rectangular areas of 0.06 m^2^ (printer paper sheet) were identified by placing adjacent printer paper sheets next to a corner on a large surface such as the tables. One 0.06 m^2^ area was sampled from each flat surfaces such as the tables, the window bar, and the floor; non-flat surfaces were sampled by swabbing the surface as completely as possible. Since only the tables were cleaned at the beginning of the month, the same areas on the fixed and movable tables were sampled during both the first and second sampling period to test for THC accumulation occurred between the two sampling period. Three wipes were used for the same area on each surface tested. The following swabbing patterns were used: back and forth starting from the top left corner to the bottom right corner of the area, up and down from the bottom left corner to the top right corner of the area and small circular motions starting at the center moving out. All 3 wipes were placed in the same glass vial using a bent paperclip to reduce handling, gloves and paperclips were changed after each sampling to avoid possible cross-contamination.

To test how easily THC could be removed from surfaces, a set of three surfaces of identical contingent area (0.036 m^2^) were identified on the windowsill; one was sampled using 1 swab, one using 2 swabs and one using 3 swabs. In order to blind the analytical laboratory, additional swabs were inserted in the glass vials without being used so that each sample tube contained three swabs. To test if three swabs were sufficient to completely remove THC from a surface, during the second sample collection, we swabbed twice the area located on the movable table, 10 minutes apart to allow the complete evaporation of the IPA solution.

Furthermore, negative controls were collected from three different locations: the laboratory bench after thorough cleaning with Sani-Cloth germicidal disposable wipes; the face of the clock situated outside the administration room and three different location in a non-user house.

### Quantification of THC on the Wipes using LC-MS/MS

The THC reference material and its isotope labeled internal standard Δ9-tetrahydrocannabinol-d3 (THC-d3) were purchased from Cerilliant (Round Rock, TX). Wipe specimens were analyzed using liquid chromatography-tandem mass spectrometry (LC-MS/MS). Briefly, the three wipes collected from each surfaces were soaked in the glass vials with 2 mL of methanol containing the internal standards (0.25 ng/mL). One thousand two hundred µL was collected and transferred into a 1.5 mL low binding polypropylene vial (Sarstedt, Nümbrecht, Germany). Specimens were dried overnight using a speed-vac (Centrivap, Labconco, Kansas City, MO, USA). Dried samples were reconstituted with 550 µL of methanol and briefly vortexed. After adding 450 µL of water, specimens were vortexed and centrifuged (at 26,000 g, 4 °C, 20 min, MR 23i, Thermo Scientific, Waltham, MA, USA). The supernatant was transferred into an HPLC vial for analysis. THC was analyzed by LC-MS/MS on a Sciex API5000 tandem mass spectrometer (Sciex, Concord, ON, Canada) via a turbo V ion source operated in the positive electrospray ionization (ESI) mode. The mass spectrometer operated in positive multiple reaction monitoring (MRM) mode. Monitored transitions for THC are reported in Table 1, transitions in bold were used for THC quantification. Two hundred fifty µL of the samples were injected onto a 4.6 · 12.5 mm online extraction column (Zorbax XDB C8, Agilent Technologies) with a particle size of 5 µm. Samples were loaded and washed with a mobile phase of 45% methanol supplemented with 0.1% formic acid and 55% 0.1% formic acid in water. The flow was increased from 0.5 mL/min to 1.5 mL/min within one minute. The extraction column was kept at room temperature. After 1 minute, the switching valve was activated and the analytes were eluted in the backflush mode from the extraction column onto a 4.6 · 50 mm Poroshell Eclipse C18, 2.7 µm analytical column (Agilent Technologies). The analytical column was kept at 60 °C. The organic solvent (B) consisted of 20% isopropanol, 20% methanol and 60% acetonitrile and the aqueous solvent (A) consisted of water supplemented with 0.1% formic acid. The analytical gradient started with a flow rate of 0.75 mL/min and 60% of solvent B for the first minute. Within the following 3 minutes, the flow rate and the organic solvent content were increased to 1 mL/min and 95% solvent B, respectively. From 4 to 6 minutes, the solvent B was increased to 100%. At minute 6.2 the system returned to starting conditions for 1.8 minutes to equilibrate for the following injection. The nebulizer current was set to 5 µA, the source gas 2 was set to 40 (arbitrary units), the source temperature was set to 450 °C, the entrance potential and the collision cell exit potential were set to 10 V and 11 V, respectively. Calibration curves were prepared by spiking 0.017 m^2^ of clean smooth surface with known amounts of THC (5–50 ng) resulting in an area concentration range of 295 ng/m^2^ to 2959 ng/m^2^. Lower limit of quantification (LLOQ) were establish as signal-to-noise ratio ≥8, no significant interferences and accuracy and imprecision within ±20% (n = 6). Surface recovery efficiency was calculated by comparing samples spiked on the surface and spiked directly on the swabs and swab matrix effect by comparing samples spiked on the swabs and extracted neat solutions in absence of swabs. To test how easily THC could be removed from surfaces, a set of three surfaces of identical contingent area (0.017 m^2^) were identified on the laboratory bench and spiked with 10 ng of THC; one was sampled using 1 swab, one using 2 swabs and one using 3 swabs. In order to simulate the experimental conditions, additional swabs were inserted in the glass vials without being used so that each sample tube contained three swabs.

**Table 1:** LC-MS/MS parameters for THC and internal standard on surfaces. Transition in bold was used for THC quantification.

Compound	Q1 (amu)	Q3 (amu)	DP (V)	EP (eV)	CE (V)	CXP (V)
THC	315.2	193.1	60	10	30	11
THC	315.2	259.3	60	10	28	11
THC-d3	318.4	196.3	60	10	30	11

DP: declustering potential, EP: exit potential, CE: collision energy, CXP: collision cell exit potential; amu: atomic mass unit; THC: δ^9^-tetrahydrocannabidiol.

Analyst software version 1.6.2 was employed for data acquisition and MultiQuant version 2.1.1 for data analysis (Sciex, Foster City, CA, USA). THC concentrations were quantified based on the THC/internal standard ratios. The calibrator were fitted using a linear equation in combination with 1/x weighting.

## Results

**Figure 1:**
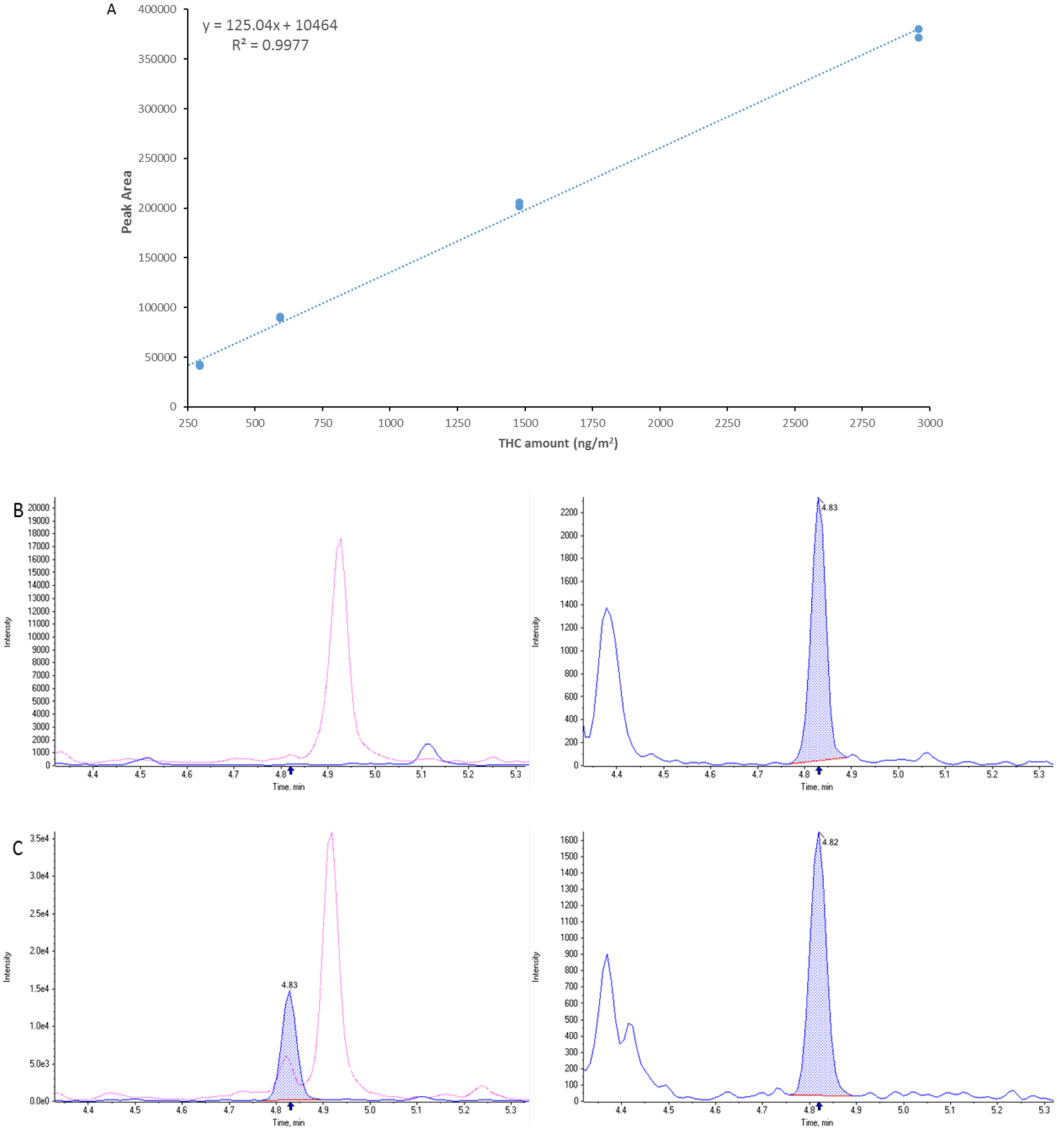
(**A**) representative calibration curve. The assay was linear from 295 ng/m^2^ to 2959 ng/m^2^. (**B**) shows a representative ion chromatogram of a negative sample and (**C**) a representative ion chromatogram of a positive sample (2024 ng/m^2^). Right chromatogram THC, blue dotted area represents the quantifier transition and the red dotted line represents the qualifier ion transition. The left ion chromatogram shows the internal standard THC-d3.

Methanol, acetonitrile and isopropanol, as well as different kind of swabs were tested during method development and achieved similar recoveries. Novaplus IPA imbued swabs were chosen as these were easily commercially available whereas methanol was selected as extraction solvent due to easier evaporation in the speed-vac. Recovery from surfaces was 63.5% ± 8.31% (n = 3) and no significant matrix interferences were noted. Absolute matrix effect of the swab extracts was 6.58% ± 0.51% and the relative matrix effect was 9.88% ± 1.02% (n = 3). The method achieved excellent linearity (r = 0.997) over the area concentration range. Lower limit of quantification was 295 ng/m^2^ and upper limit of quantification was 2959 ng/m^2^ for THC (n = 3) (Fig. 1A). THC concentrations for samples above the upper limit of quantification were estimated based on extrapolation of the calibration curve above the highest calibrator. Samples exceeding the ULOQ were generated to prove applicability of curve extrapolation and back-calculated values were within ±20%. Since the three swabs were extracted together, THC concentrations were reported as the mean of the three swabs. Back-calculated values for samples obtained by spiking 10 ng of THC on 0.017 m^2^ and collected using 1, 2 or 3 swabs gave similar results.

THC was detected on 6 out of the 15 tested surfaces at levels greater than the lower limit of quantification (Table 2, Fig. 1B,C). All four negative control samples and laboratory blanks tested negative. Samples collected from the floor, the doorknob and the window bar tested below the lower limit of quantification, as well as the sample from the fixed and the movable table collected during the first sampling period. THC was detected in the samples collected during the second sampling period from the same areas on the fixed and movable table (2339 ng/m^2^ and 2024 ng/m^2^, respectively). The second sample collected from the movable table after 10 minutes tested positive for THC (843 ng/m^2^). Samples collected from the window sill using only 1 swab or 2 swabs tested below the lower limit of quantification. The highest level of THC (4882 ng/m^2^) was measured on the windowsill in the drug administration room that was swabbed 3 times and had not been cleaned by study personnel during the study. THC was detected even in the samples from the sides of the Volcano vaporizer (349 ng/m^2^) and from the seat of the reclining chair used by the study participant (703 ng/m^2^). The Grooved Pegboard Task assessment used during the study tested negative during the first sampling period but was positive in the second sampling period (997 ng/m^2^).

**Table 2:** THC amounts detected on different surfaces exposed to cannabis vapors.

Sample Description	Surface Area (m^2^)	THC (ng/m^2^)
**First Sampling Period**		
Floor	0.06	BLQ
Chair Seat	0.31	704
Fixed Table	0.06	BLQ
Movable Table	0.06	BLQ
Grooved Pegboard Task assessment	unknown	BLQ
Door Knob	0.012	BLQ
Volcano	0.034	349
Window Bar	unknown	BLQ
**Second Sampling Period**		
Fixed Table	0.06	2339
Movable Table Sample 1	0.06	2024
Movable Table Sample 2*	0.06	843
Window Sill x 1 Swabs	0.036	BLQ
Window Sill x 2 Swabs	0.036	BLQ
Window Sill x 3 Swabs	0.036	4882**
Grooved Pegboard Task assessment	unknown	997
**Negative Control Surfaces**		
Clock Face	0.06	BLQ
Home Location 1	0.06	BLQ
Home Location 2	0.06	BLQ
Home Location 3	0.06	BLQ

Hard surfaces present in the study room were sampled using isopropanol imbued non-woven wipes. One area was sampled from each surface tested. Unless otherwise stated, each surface was swabbed three times following a standardized protocol including three different swabbing patterns. Negative samples were collected following the same protocol from the clock outside the study room and from different locations in the house of a non-user.

BLQ: below limit of quantification; *sample collected from the same area after 10 minutes; **estimated value above upper limit of quantification.

## Discussion

Delgado-Rendon *et al*. showed that a significant portion of the general population is not aware of the health risks associated with second or third hand exposure to cannabis^21^. Literature is available on the effects of second-hand exposure to cannabis smoke in exposed non-user subjects although no research is available on the secondhand exposure effects of vaping^7–9^. Furthermore, some studies^5,12,22^ considered environmental exposure by testing air cannabinoids content but none, to our knowledge, have considered the possibly contaminated surfaces as a source of third-hand exposure^11,23^. In recent years, tobacco research showed that house dust and surfaces are responsible for third-hand exposure by non-smokers and children^17,24^. In the present analysis, we demonstrated that detectable levels of THC were recovered from surfaces in a drug administration room used by an ongoing study that includes vaporizing cannabis. As most aspects of drug administration were known, the study room provided a well-controlled environment for sampling surfaces and measuring cannabinoid contamination after vaporization. The study room was equipped with a ventilation system that was designed to completely exchange room air within two minutes and the vaporizer balloon was designed to limit the release of vapor into the environment. Such an efficient ventilation system is not common in real-life situations, suggesting that accumulation of THC on surfaces by side-stream smoke or vapor will be even higher in homes of cannabis users than under these controlled conditions.

We collected samples from nine different surfaces throughout the room and we focused on hard surfaces that could not be damaged by IPA; further studies are needed to test possible accumulation of porous surfaces and/or cloths. We attempted to reduce possible cross-contamination by collecting field blank samples, placing the swabs in the container with a bended paperclip and changing paperclips and gloves after every sampling. Most of third-hand exposure tobacco researchers use cotton wipes imbued with water or 1% ascorbic acid to collect environmental samples for nicotine analysis^16,17^. Cannabinoids are less water soluble and more lipophilic, so we used swabs imbued with water and IPA (30:70, v/v); although, IPA can damage specific surfaces (i.e., wall paint).

In the present study, the vaporizer was loaded with cannabis product prior to being brought into the room so positive samples were not due to handling of the cannabis product itself. Furthermore, the high THC level measured on the windowsill suggested deposition of vapors or cross-contamination. Interestingly, only the surfaces swabbed three times, that was farther away to the ventilation system, showed positive results supporting the hypothesis of vapor deposition or contamination due to specific air circulation patterns. Indeed, there was no difference in the results when a surface was spiked with known amount of THC and swabbed 1, 2 or 3 times in the laboratory settings. This observation may represent a public health message as there are no guidelines for people using cannabis in their homes on how to clean surfaces in a way that removes the possibility of third hand contamination for other people living in the home. Indeed vaporizing cannabis is generally advertised as a safer alternative to smoking for the user as it creates less byproducts compared to combustion and this method continues to grow in popularity^3,4^. It is clear from this study that vaporizing cannabis in a controlled way (by collecting the vapor in a bag designed to contain it) and in a controlled environment (with an exhaust system) results in environmental contamination by THC implying that cannabis used in the home in a less controlled way will at least have a similar effect. These findings support the hypothesis that exposure to a contaminated environment might result in detection of cannabinoids in hair samples even if the subject is not present during smoking/vaping episodes^14,20^. This observation may help the interpretation of cannabinoid findings in hair samples in forensic toxicology cases such as child-custody cases when the child lives in a contaminated environment.

The same areas on the fixed and movable tables were sampled during both the first and second collection and only the second sets of samples were positive for THC. A possible explanation is the cleaning schedule by the study staff. The first collection happened at the beginning of the month, shortly after the study staff cleaned the tables with IPA cloths but the tables were not cleaned before the second collection allowing the deposition of THC from the 6 study visits that happened in between. Interestingly, the same area sampled twice during the second sampling period tested positive both times suggesting that more wipes might be needed to completely remove the THC.

The present study has several limitations and strengths. Limitations include a limited sample number, a limited number of experimental replicates and the inability to know whether each specific session used active or placebo cannabis. However, the study protocol is fixed and the number of participants who completed all the sessions is known creating boundaries for the number of known exposures that occurred before each sampling period. The sampling protocol mimics that used for other environmental drug exposure assessments and sample handling, storage and laboratory methodologies are appropriate and reduce contamination and error. Furthermore, we were able to detect quantifiable THC levels in presence of an excellent ventilation system that is intended to reduce secondhand exposure to cannabis. The constant removal of vapor from the room might have affected our data causing an underestimation compared to a real-life environment where such efficient ventilation systems are not common. These are the first data showing that cannabis side-stream vapor contaminates surfaces and is a potential source for third-hand user and bystander exposure. Further studies are needed to better understand the deposition of cannabis smoke or vapor in real-life environments, how THC cannabis potency and consumption method affect the contamination of surfaces and whether and how THC deposited on surfaces can be transferred to subjects for later ingestion or for deposition on hair. Lastly, this study suggests that a cleaning protocol should be developed to aid cannabis users in cleaning surfaces that might become a route of tertiary exposure to other people living in their home.

## Conclusions

We showed that in a room in which cannabis was administered by vaporization surfaces tested positive for THC at quantifiable levels. This study represents a first step in understanding how side-stream cannabis vapor deposits in the environment and may result in tertiary exposure to users and bystanders.
